# The Role of Matrix Metalloproteinase in the Intimal Sarcoma-Like Cells Derived from Endarterectomized Tissues from a Chronic Thromboembolic Pulmonary Hypertension Patient

**DOI:** 10.1371/journal.pone.0087489

**Published:** 2014-01-28

**Authors:** Takayuki Jujo, Seiichiro Sakao, Masanori Tsukahara, Seiji Kantake, Miki Maruoka, Nobuhiro Tanabe, Masahisa Masuda, Koichiro Tatsumi

**Affiliations:** 1 Department of Respirology (B2), Graduate School of Medicine, Chiba University, Inohana Chuo-Ku, Chiba, Japan; 2 Respirology, Kimitsu Chuo Hospital, Sakurai, Kisarazu City, Japan; 3 Respirology, National Hospital Organization Chiba Medical Center, Tsubakimori, Chuo-ku, Chiba, Japan; 4 Cardiovascular Surgery, National Hospital Organization Chiba Medical Center, Tsubakimori, Chuo-ku, Chiba, Japan; Keio University School of Medicine, Japan

## Abstract

Sarcoma-like cells (SCLs) were derived from endarterectomized tissue of a single chronic thromboembolic pulmonary hypertension (CTEPH) patient during incubation of those thrombi at second passage as described at our previous report. These cells had malignant potential, with an increased expression of matrix metalloproteinase-14 (MMP-14), leading to tumor emboli within pulmonary arteries in *in vivo* studies. The purpose of this study was to perform a more detailed evaluation of the characteristics of SCLs, and to elucidate the role of the increased expression of MMP-14 expression in the growth and death of these cells. In order to elucidate the characteristics of SCLs and to confirm the protein expression of MMP-14, three-dimentional culture, invasion assays, a Western blot analysis and immunohistochemical studies were performed. To examine the role of MMP-14 in tumorigenesis, the metalloproteinase inhibitor, batimastat, was administered to SCID mice which were subcutaneously injected with SCLs. Those mice were sacrificed on day 14 and the tumor volume was evaluated. A Western blot analysis showed the increased expression of MMP-14 in comparison to the expression in lung adenocarcinoma cells (A549). Immunohistochemistry showed that SCLs were positive for vimentin, MMP-14, MMP-2 and CD44. However, endothelial markers, such as CD31 and von Willebrand factor (vWF), were negative. The *in vivo* studies demonstrated that batimastat could suppress the growth of the subcutaneous tumors formed by the SCLs. This study suggested that MMPs had critical roles on the pathological activities of SCLs and that batimastat might have anti-proliferative and anti-invasive effects on these cells.

## Introduction

The organized thrombi of chronic thromboembolic pulmonary hypertension (CTEPH) are composed of several cell phenotypes. Some populations have high biological activities [1], and some are positive for α-smooth muscle actin (α-SMA), which were considered to be “myofibroblast-like cells” from the endarterectomized tissues of the CTEPH patient [1], [2], [3]. We previously showed that the myofibroblast-like cells were hyperproliferative, invasive and anchorage-independent [3], and these characteristics are considered to be cancer hallmarks [4]. We also showed that sarcoma-like cells (SCLs) derived from a single CTEPH patient could be isolated at the second passage of the myofibroblast-like cells, and these had an increased expression of matrix metalloproteinase-14 (MMP-14), and had high tumorigenic potential to form solid and undifferentiated tumors which grew along the intimal surface of the pulmonary arteries in C.B-17/lcr-scid/scidJcl mice [5]. These results suggested that the behavior of SCLs closely resembled that of pulmonary intimal sarcoma. Pulmonary intimal sarcoma is a very rare mesenchymal neoplastic tumor [6] that is highly resistant to treatments, including anti-cancer drugs.

The purpose of this study was to obtain more details about the characteristics of SCLs in comparison to A549 epithelial cancer cells, and to elucidate the role of the increased expression of MMP-14 in the growth and death of the cells. This is the further report focusing on MMP-14 following our previous report [5]. The results of this study may lead to the development of a new therapeutic approach for this uncommon sarcoma.

## Materials and Methods

### Ethic statement

All procedures performed in this study were approved by the Research Ethics Committee of Chiba University School of Medicine and Chiba University Instrumental Animal and Use Committee. Written informed consent was given by all subjects.

### Clinical presentation of the patient

SCLs were derived from a single patient. The patient was a 64 years-old man with a history of acute pulmonary embolism and consulted to a hospital because of hemoptysis, which was treated by a bronchial artery embolization (BAE) procedure. The results of detailed examinations diagnosed him as pulmonary hypertension and pulmonary arterial embolism. Therefore, he was referred to our hospital. Preoperative hemodynamic data was as follows, mean pulmonary arterial pressure (mPpa); 55 mmHg, pulmonary vascular resistance (PVR); 982 dyne sec cm^−5^. By computed tomographic (CT) scan, lung perfusion scan, and pulmonary angiography, chronic pulmonary embolism was detected. He was diagnosed as CTEPH and pulmonary endarterectomy (PEA) was performed by Dr. Masahisa Masuda at the Chiba Medical Center, Japan. After the surgery, the hemodynamic improved as follows, mPpa; 17 mmHg, PVR; 202 dyne sec cm^−5^. Resected organized thrombi were investigated pathologically. Pathological diagnosis was atherosclerosis-intima fibrosis of pulmonary arteries with partial recanalization, which was the typical finding of chronic pulmonary thrombosis, and no malignant cell was detected. Although it's already been five years or more after the operation, he has no intimal sarcoma now.

### Cell isolation

One part of resected organized thrombi was investigated pathologically and the other part was done in this study. At the second passage of the myofibroblast-like cells obtained following incubation of the endarterectomized tissues, pleomorphic cells (called sarcoma-like cells (SCLs)), were isolated, probably by chance. The details of the cell isolation have been described previously [5].

### Cell lines and reagents

A549 cells (a human lung carcinoma cell line) were purchased from Takara Biomedical (Ohtsu, Shiga, Japan). SCLs were incubated using endothelial cell growth medium supplemented with 5% fetal bovine serum (FBS) and growth factors (EGM-2) (Lonza Inc, Allendale, NJ, USA) at 37°C in 5% CO_2_ in air in a humidified incubator. A549 cells were cultured using RPMI 1640 media (Life Technologies, Carlsbad, CA, USA) with 5% FBS. In order to exclude the effects of several growth factors present in the EGM, endothelial cell basal medium-2 (EBM-2) (Lonza Inc, Allendale, NJ, USA), with only 5% FBS, was used in some experiments. Batimastat, a synthetic matrix metalloproteinase inhibitor, was purchased from Merck Millipore (Darmstadt, Germany). Batimastat was diluted in dimethyl sulfoxide, stored at −20°C and was prepared just before use in experiments. The antibodies used for the immunohistochemical studies were: mouse anti-vimentin (1∶200, Dako, Carpinteria, CA, USA), mouse anti-human, rabbit anti-von Willebrand factor (factor VIII) (1∶1,000, Dako), rabbit anti-CD31 (1∶200, Abcam, Cambridge, UK), anti-MMP-14 (1∶200), rabbit anti-MMP-2 (1∶200, Abcam), mouse anti-CD44 (1∶100, Abcam), an anti-mouse IgG conjugated with Alexa-488 fluorescent dye (1∶200, Molecular Probes, Tokyo, Japan), an anti-rabbit IgG conjugated with Alexa-488 fluorescent dye (1∶200, Molecular Probes), rabbit anti-MMP-14 (1∶200, Abcam), MMP-2 (1∶100, Abcam) and polyclonal biotinylated goat anti-rabbit immunoglobulins (1∶500, Dako). The antibodies used in the Western blot analysis were: MMP-14 antibodies (1∶500, Abcam), MMP-2 antibodies (1∶500, Abcam), β-actin antibodies (1∶1000, Cell Signaling Technology, Boston, MA, USA) and stabilized goat anti-rabbit IgG (H+L), which was peroxidase conjugated (ThermoScientific, Massachusetts, MA, USA).

### PCR array

In order to confirm the different RNA expression levels between SCLs as mesenchymal malignant tumor cells and A549 as epithelial cancer cells, a PCR array analysis focusing on the adhesion molecules was performed. Our previous study had demonstrated that there was increased mRNA expression of MMPs in SCLs in comparison to A549 cells [5]. Therefore, a PCR array was used to confirm the precise expression levels of various MMPs. The RT^2^ Profiler PCR Array (SABiosciences, Frederick, USA) was used for this purpose. The details of this protocol have been described previously [5].

### Invasion and migration assay

The BD BioCoat FluoroBlok Invasion System (24-multiwell) was used to assess the invasion and migration of SCLs with or without batimastat treatment. In order to exclude the effects of additives, including growth factors, SCLs were incubated with the EBM-2 for three days. Then, 2.5×10^5^ pretreated SCLs were seeded to each upper well of the kit with serum free medium. The medium added to each lower well was as follows: EBM-2 containing 5% FBS and dimethyl sulfoxide (the same quantity as the solvent used for dissolving 10 µM batimastat) (control group), 5% FBS and 1 µM batimastat (1 µM batimastat group) or 5% FBS and 10 µM batimastat (10 µM batimastat group). The kits were incubated at 37°C in 5% CO_2_, in a humidity-controlled incubator for 16 hours. The fixation, staining and quantification of the invaded cells were described previously [5].

### Three-dimensional cultures

BD Matrigel™ Basement Membrane Matrix Growth Factor Reduced (BD Falcon, USA) was used for three-dimensional cultures. This experiment was conducted in accordance with the recommended “thin-gel method”. Matrigel was gently thawed in a 4°C refrigerator. A 50 µl aliquot of Matrigel was added thinly to each wells of a 24-well plate. The plates were incubated in a 37°C incubator for 15 minutes. After that, 20 µl of Matrigel and 5×10^4^ cells suspended in 1000 µl medium were mixed and seeded into each well. The findings in SCLs and A549 were investigated. In order to study the effects of batimastat, 1 µM or 10 µM of batimastat were added to the EBM-2 media. Finally, the plates were incubated at 37° in a 5% CO_2_ in air in a humidified incubator for different periods of time.

### Western blot analysis

The cultured cells on the dishes were washed, homogenized in lysis buffer (20 mM Tris-HCl, pH 8.0, 1 mM EDTA, 1 mM NaN_3_, 1 mM DTT, 150 mM NaCl, 0.5% Triton-X, phosphatase inhibitor cocktail (SIGMA P5726)), and centrifuged at 10,000×g for 5 min. The protein concentrations of these supernatants were measured by the Bradford method (Bio-Rad protein assay; Nippon Bio-Rad Laboratories, Tokyo, Japan). Protein samples (2 µg) were separated on 10% Tris-glycine gels (Invitrogen Japan, Tokyo, Japan) and transferred to nitrocellulose membranes (Invitrogen Japan, Tokyo, Japan). Membranes were blocked with 5% non-fat dried milk in PBS containing 0.5% Tween20 for 1 hour at 4°C, and were then incubated with primary antibodies. The membranes were incubated with peroxidase-conjugated secondary antibodies for 1 hour at room temperature. Chemiluminescence was detected by a LAS-1000 instrument (Fuji Film, Tokyo, Japan). Signals were quantified using the Fuji Image Gauge software program (ver. 3.0, Fuji Film, Tokyo, Japan).

### Cell culture with a matrix metalloproteinase inhibitor

SCLs were incubated with growth factor-free medium for three days. They were then washed and trypsinized. A total of 1×10^5^ cells were added to fibronectin-coated dishes. They were incubated with the EBM-2 containing DMSO (control group), 1 µM batimastat (1 µM batimastat group) or 10 µM batimastat (10 µM batimastat group) at 37° in a 5% CO_2_ in air in a humidified incubator for 24 hours, 48 hours or 72 hours. At the appropriate times, the cell numbers were counted.

### Intravascular and subcutaneous tumor formation

Icr/scid mice were purchased from Nihon Clea. SCLs incubated in EGM media were trypsinized, and 2×10^6^ cells were injected intravenously into the mice. Twenty-eight days after injection, the mice were sacrificed, and various organs, including the lungs, were resected. These tissues were pathologically investigated. A total of 1×10^6^ cells were injected subcutaneously into the Icr/scid mice. Then 40 mg/kg batimastat was injected intraperitoneally once a day from day 3 to day 13. On day 14, they were sacrificed, and the subcutaneous tumors were resected. Tumors were weighed and pathologically examined.

### Immunohistochemistry

Samples were fixed in 10% buffered formalin, paraffinized and sliced at 1.5 µm thick. Antigen retrieval was performed using pH 6.0 citrate buffer (Abcam # 64214) for the deparaffinized slices. Sections were blocked with 2% normal goat serum, PBS(-) and 0.1% Tween20 for 30 min at room temperature. They were then incubated with the primary antibodies for 1 hour at 4°C and with secondary antibodies for 30 minutes at room temperature. The avidin-biotin-peroxidase complex method with peroxidase streptavidin (Nichirei #426062, Tokyo, Japan) and the DAB substrate kit (Abcam ab64238) was performed.

### Statistical analysis

The PCR array data were analyzed by a web-based software program, the RT^2^ Profiler PCR Array Data Analysis, version 3.5 (http://pcrdataanalysis.sabiosciences.com/pcr/arrayanalysis.php). The other data were analyzed using the useful and reliable statistical software, EZR on R commander [7] (ver.1.03, provided on the site of Division of Hematology, Saitama Medical Center, Jichi Medical University (http://www.jichi.ac.jp/saitama-sct/SaitamaHP.files/statmedEN.html)). At least three samples were used for the statistical analyses. We considered that the differences were significant for values of p<0.05.

## Results

### MMPs expression

**Figure 1:**
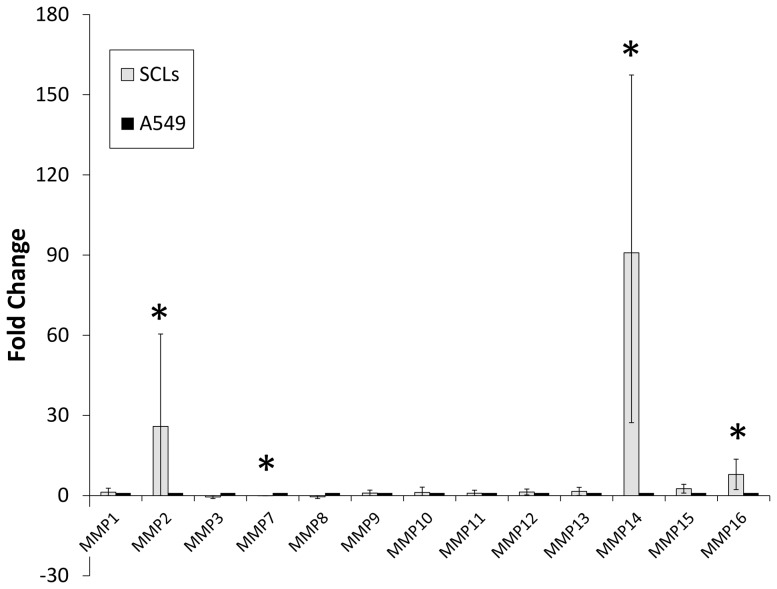
The results of the PCR array analysis focused on the matrix metalloproteinases (MMPs) of SCLs. The expression levels of MMP-14 and MMP-2 of SCLs were higher than those of A549 cells, while the level of MMP-7 was lower. These data were analyzed by a web-based software program (the RT^2^ Profiler PCR Array Data Analysis, version 3.5). Error bars showed 95% Confidencial intervals. (* p-value <0.05 versus A549, n = 5)

**Figure 2:**
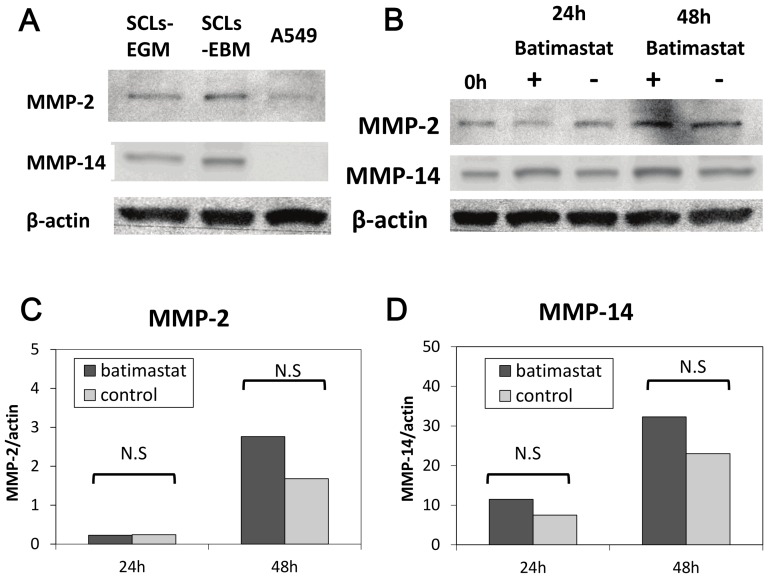
The results of the Western blot analysis. (A) The Western blot analysis revealed that the MMP-14 protein expression in SCLs was higher than that in A549 cells. There was no significant difference in the protein expression of MMP-14 in SCLs incubated in the media with or without growth factors. (B) Batimastat did not suppress the SCLs' expression of the MMP-14 and MMP-2 proteins. (C)(D) The band signal strength of MMP-14 (c) and MMP-2 (d) expressed as a ratio to beta-actin. No significant differences in the signal strength were recognized 24 hours and 48 hours after batimastat exposure between the batimastat and control groups. (“N.S.” showed no significant difference between two groups analyzed by Student's t-test.)

The PCR array analysis showed that there was increased mRNA expression for MMP-2, MMP-14 and MMP-16 in the SCLs in comparison to the A549 cells (Table 1, Figure 1). In addition, decreased expression of MMP-7 mRNA was confirmed in SCLs in comparison to the A549 cells (Table 1, Figure 1). A Western blot analysis revealed that the MMP-14 protein expression in SCLs was higher than that in A549 (Figure 2A). There was no obvious difference in the protein expression of MMP-2 and MMP-14 in the SCLs incubated in the media with (EGM) or without (EBM) growth factors (Figure 2A).

**Table 1:** Expressions of matrixmetalloproteinase of SCLs on PCR array.

Description	Gene symbol	Public ID	Fold Change (95%CI)	p-value
Matrix metallopeptidase 1 (interstitial collagenase)	MMP1	NM_002421	1.2872 (0.00001–2.77)	N.S.
Matrix metallopeptidase 2 (gelatinase A, 72 kDa gelatinase, 72 kDa type IV collagenase)	MMP2	NM_004530	25.9049 (0.00001–60.45)	<0.05
Matrix metallopeptidase 3 (stromelysin 1, progelatinase)	MMP3	NM_002422	−0.4998 (−1.10– −0.00001)	N.S.
Matrix metallopeptidase 7 (matrilysin, uterine)	MMP7	NM_002423	−0.037 (−0.06– −0.01)	<0.00005
Matrix metallopeptidase 8 (neutrophil collagenase)	MMP8	NM_002424	−0.4566 (−1.11– −0.00001)	N.S.
Matrix metallopeptidase 9 (gelatinase B, 92 kDa gelatinase, 92 kDa type IV collagenase)	MMP9	NM_004994	0.9158 (0.00001–2.04)	N.S.
Matrix metallopeptidase 10 (stromelysin 2)	MMP10	NM_002425	1.1935 (0.00001–3.16)	N.S.
Matrix metallopeptidase 11 (stromelysin 3)	MMP11	NM_005940	0.8688 (0.00001–2.01)	N.S.
Matrix metallopeptidase 12 (macrophage elastase)	MMP12	NM_002426	1.3401 (0.24–2.44)	N.S.
Matrix metallopeptidase 13 (collagenase 3)	MMP13	NM_002427	1.5681 (0.05–3.09)	N.S.
Matrix metallopeptidase 14 (membrane-inserted)	MMP14	NM_004995	90.8284 (27.28–154.38)	<0.01
Matrix metallopeptidase 15 (membrane-inserted)	MMP15	NM_002428	2.573 (0.93–4.22)	N.S.
Matrix metallopeptidase 16 (membrane-inserted)	MMP16	NM_005941	7.9291 (2.24–13.62)	<0.05

### Immunohistochemistry

**Figure 3:**
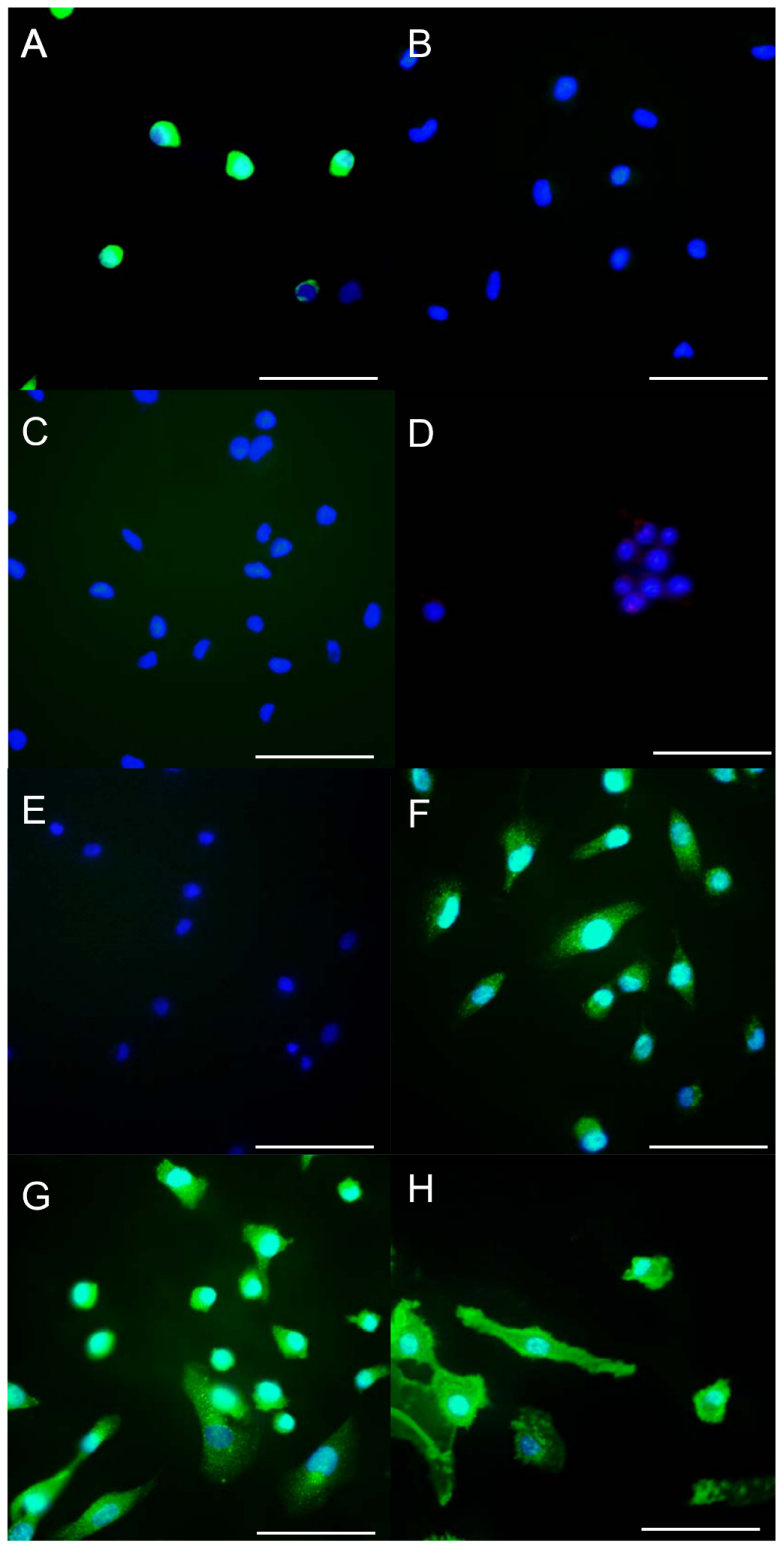
The results of the immunohistochemical analysis of the SCLs. The SCLs were positive for vimentin (A), MMP-14 (F), MMP-2 (G) and CD44 (H). In contrast, the staining for α-SMA (B), VWF (C), CD31 (D) and desmin (E) was negative. (Scale bars show 50 µm.)

**Figure 4:**
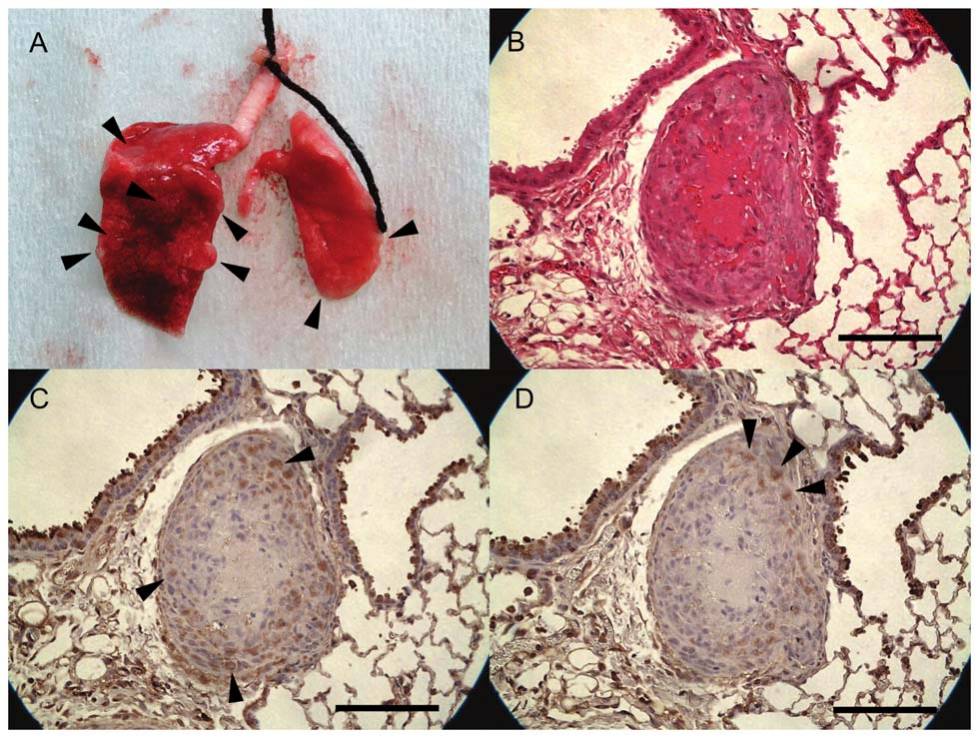
Tumors which grew along the intimal surface of the pulmonary arteries. (A) A resected lung from a mouse. SCID mice were sacrificed 28 days after the injection of SCLs. Many nodules were located on the surface of the lungs. Arrow heads showed some of these nodules. (B) HE staining showed that the tumors were composed of a central area with necrosis and a peripheral zone filled with SCLs. (C) Immunohistochemical staining for MMP-14 revealed that the peripheral area of tumors was positive for MMP-14, and the central necrotic area was negative for MMP-14. (D) Immunohistochemical staining for MMP-2 showed that MMP-2 was weakly positive in the peripheral zone. (Scale bars show 100 µm.)

Immunohistochemical staining showed that SCLs were positive for vimentin (Figure 3A), MMP-14 (Figure 3F), MMP-2 (Figure 3G) and CD44 (Figure 3H) which is a cell-surface glycoprotein involved in cell adhesion and migration. However, endothelial cell markers, including Factor VIII (Figure 3B) and CD31 (Figure 3C), a smooth muscle cell marker, α-SMA (Figure 3D) and desmin (Figure 3E) were negative. C.B-17/lcr-scid/scidJcl mice which were intravenously injected with SCLs developed tumors which grew along the intimal surface of the pulmonary arteries (Figures 4A, 4B). The peripheral lesions of tumors seemed to be a cell-rich layer, while the center of the tumors was a necrotic zone (Figure 4B). The cells in the tumor were positive for MMP-14 and MMP-2 (Figures 4C, 4D).

### Three-dimensional culture

**Figure 5:**
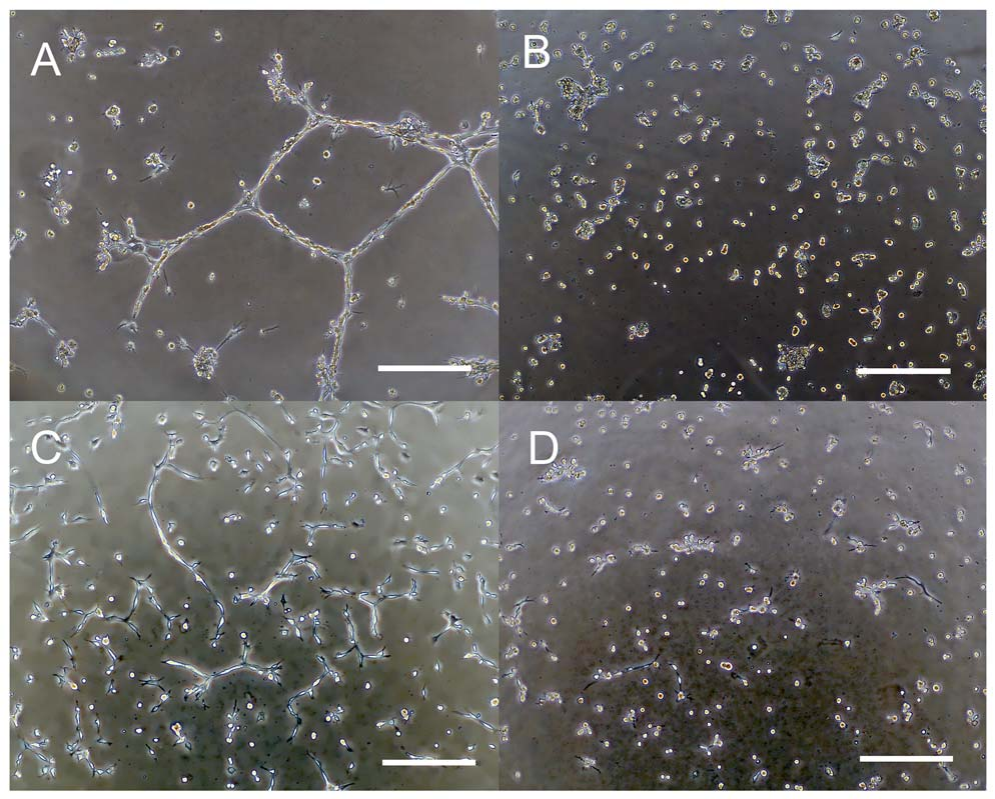
The findings of the three-dimensional culture of SCLs. Cells were incubated for 12(A and B) or 24 hours (C and D). SCLs proliferated linearly and formed tube-like structures and network-like structures within Matrigel (A), but the A549 (lung adenocarcinoma) cells formed no network structures (B). SCLs incubated with EBM-2 also formed the tube-like structures in the Matrigel (C). Exposure to 10 µM batimastat inhibited the formation of the three-dimensional structures (D). (Scale bars show 300 µm.)

The three-dimensional culture system was employed to confirm the SCL proliferation under *in vivo* conditions, which could make the cells grow in an environment that more closely resembled their normal condition. SCLs, as mesenchymal neoplastic cells, formed aligned structures as tube-like networks during the 12-hour incubation period (Figure 5A). In contrast, the A549 adenocarcinoma cells did not organize themselves into aligned structures (Figure 5B).

### Effects of a synthetic matrix metalloproteinase inhibitor, batimastat

**Figure 6:**
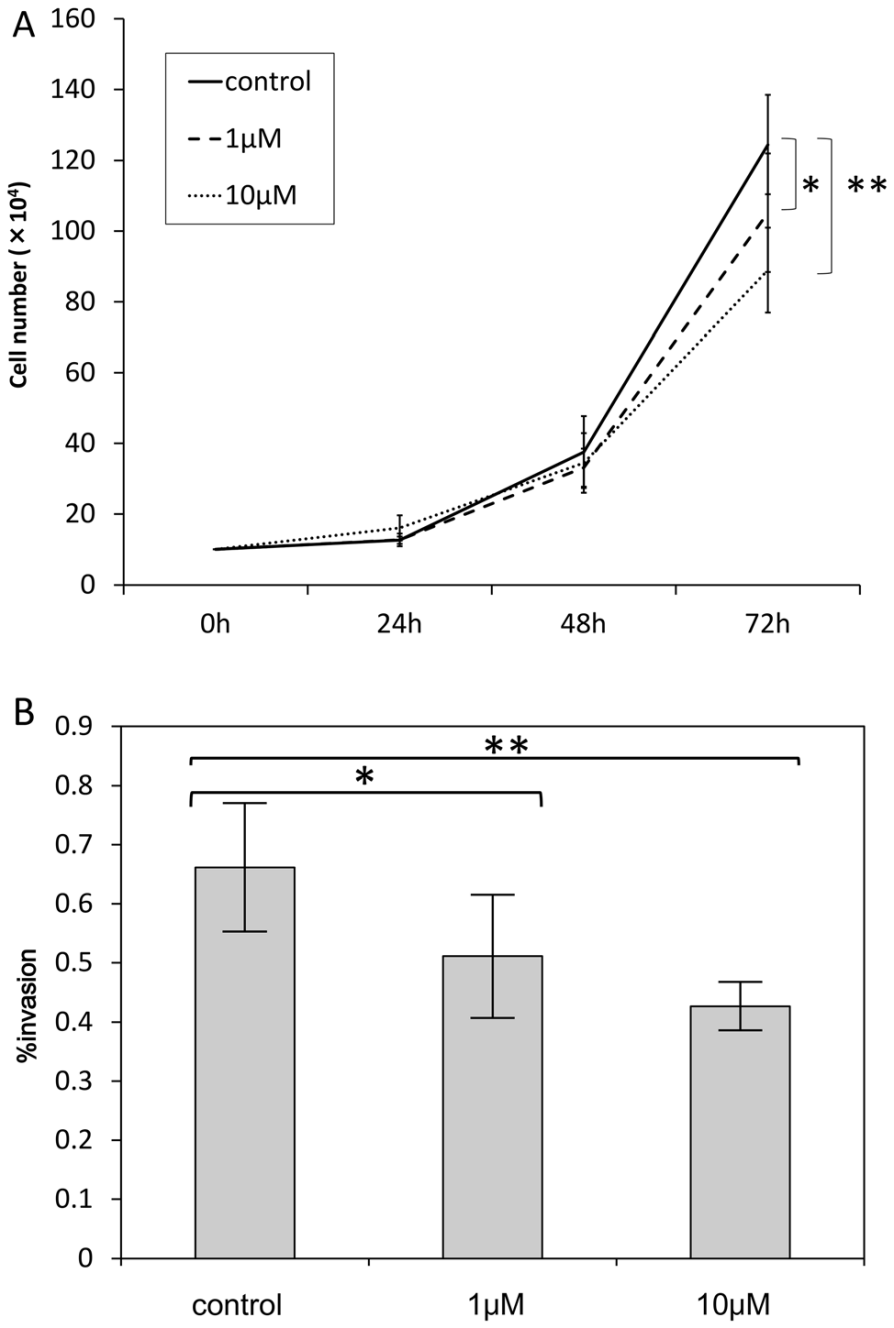
Batimastat suppressed the biological activities of SCLs in *in vivo* studies. (A) Batimastat, a synthetic matrix metalloproteinase inhibitor, suppressed the proliferation of SCLs 72 hours after incubation with batimastat. The number of SCLs that survived following the culture with batimastat was lower than that in the control group. (* p-value <0.05, **p-value<0.01 versus control group, by Student's t-test, n = 6). (B) The invasion assays revealed that the SCLs incubated with batimastat were less invasive than those in the control group. (* p-value <0.05 versus control group, **p-value<0.01 versus control group, by Student's t-test, n = 8)

In order to confirm the role of MMPs in the growth and death of SCLs, the cells were incubated in the EBM-2 media with batimastat. Although batimastat did not suppress the protein expression of MMP-14 or MMP-2 (Figures 2B-2D), this reagent did suppress the number of proliferating SCLs (Figure 6A). Moreover, the invasion assay demonstrated that batimastat decreased the invasion of SCLs (Figure 6B), and the three-dimensional culture showed that it prevented these cells from organizing themselves into aligned tube-like structures (Figures 5C, 5D).

**Figure 7:**
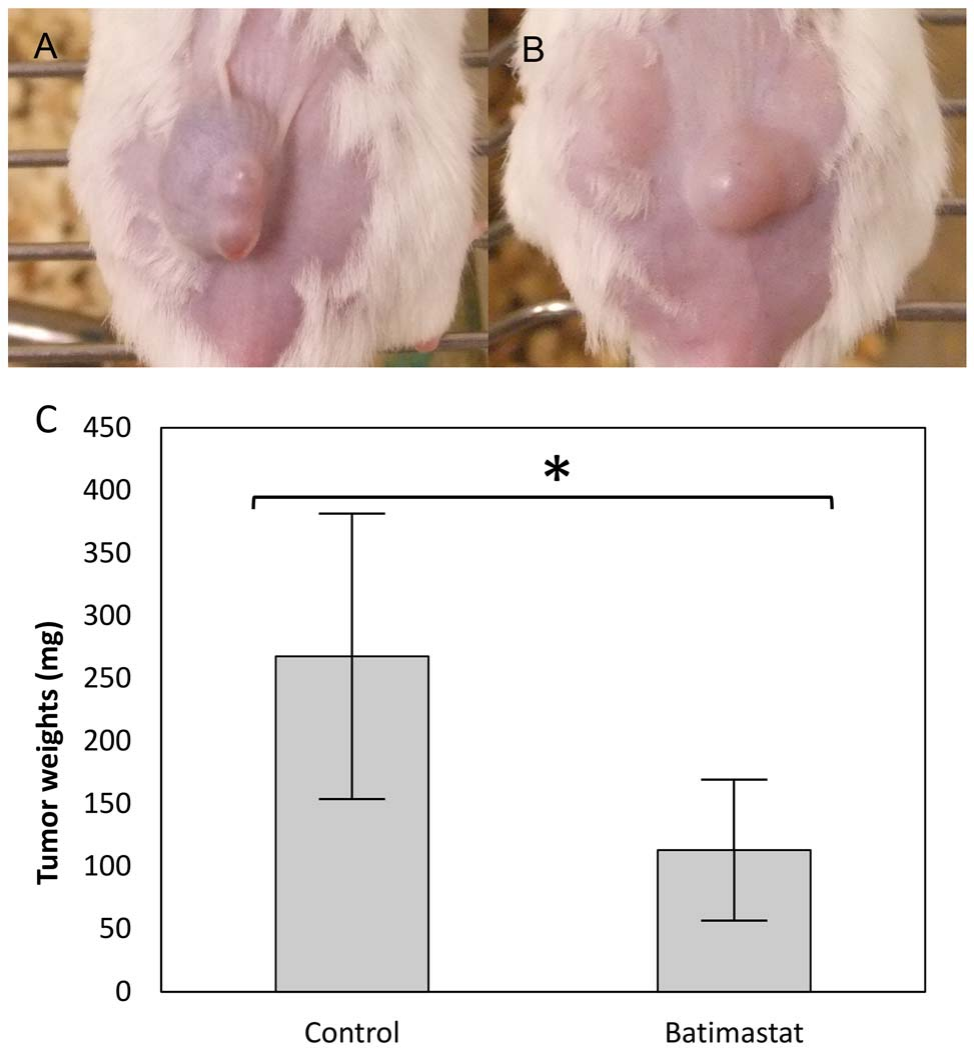
Batimastat suppressed the growth of SCLs in xenograft models. Subcutaneous tumors were formed on the backs of the mice in the control group (A) and batimastat group (B). It was revealed that there were significant differences in the tumor weights between the control group (n = 6) and batimastat group (n = 4) (C). (* p-value <0.05, versus control group, by Student's t-test)

To investigate the effects of batimastat on the tumorigenesis of SCLs in Icr/scid mice, 1×10^6^ cells were injected subcutaneously, and were then treated intraperitoneally with 40 mg/kg batimastat once a day from day 3 to day 13. On day 14, the resected subcutaneous tumors were weighed, and there was a significant difference between the control (n = 6) (Figure 7A) and batimastat (n = 4) (Figure 7B) groups (control group 267.5±113.8 mg vs batimastat group 112.9±56.4 mg, p<0.05) (Figure 7C). There were no significant differences in the body weight between the groups on the day of SCLs injection (day 0: control group 20.6±1.0 g vs batimastat group 21.7±0.8 g, p>0.05) or on the first day of batimastat injection (day 3: control group 21.7±0.9 g vs batimastat group 22.3±0.8 g, p>0.05). On day 14, there was a significant difference in the body weight between the groups (control group 20.7±0.8 g vs batimastat group 22.2±0.7 g, p<0.05).

## Discussion

The present study demonstrated that there was an increased expression of MMP-14 in SCLs (Figures 1–4) and that the blockade of the MMPs cascades by batimastat suppressed the pathobiological activities of SCLs in *in vivo* and *in vitro* experiments (Figures 5–7). MMPs are a group of endopeptidases biochemically characterized by their dependence on zinc at the active site [8], [9]. MMPs degrade most components of the extracellular matrix (ECM) and are considered to have an important role in various biological processes in cells, such as proliferation, apoptosis, invasion, differentiation and angiogenesis [8], [9]. Generally, MMPs are divided into two groups; secretion-type and membrane-type (MT). Because of its cell surface expression, MMP-14 is called MT1-MMP [10], [11], [12]. MMP-14 has many functions, including ECM degradation, the activation of MMP-2 and MMP-13 [11], [13] and the cleavage of cell surface receptors, such as CD44 [10], [11], [13]. Therefore, because MMP-14 is a modulator of the pericellular microenvironment [10], it is thought to have critical roles in controlling the invasive and metastatic capabilities of malignant cells [10], [11].

Batimastat is a synthetic MMP inhibitor, and the depressant action of this agent is biochemically explained by the binding of a hydroxamate to the zinc ion in the active center of MMPs [14]. In this study, batimastat inhibited the proliferation of SCLs (Figure 6A). Some studies, however, reported that batimastat did not suppress the proliferation of malignant cells [15], [16]. Zervos et al. showed that anti-proliferative effect of this drug on human pancreatic adenocarcinoma cells was apparent in a high dose of this drug [17], indicating that anti-proliferative effect of this drug might be in a dose-dependent manner. This supports our results on Figure 6. Moreover, batimastat suppressed invasive activity of SCLs in the present study. Ueda et al. also reported that the invasive fibrosarcoma cells were suppressed by 10 µM batimastat [18]. In truth, MMP-14 is supposed to play a critical role on cell invasion activity through effect as the degeneration of extracellular matrix [12], [19].

Kadono et al. reported that batimastat inhibited the formation of three-dimensional structures by fibrosarcoma cells [20], as well as their migration and adhesion [21]. In this study, SCLs formed tube-like or network-like structures in the three-dimensional culture, and batimastat prevented the formation of these structures by the SCLs (Figure 5). This result supported the findings in the previous report. However, A549 cells could not form the tube-like or network-like structures in the three-dimensional culture (Figure 5B), and these differences are likely attributable to the fact that the SCLs were mesenchymal cells which had originated from the pulmonary vasculature, while A549 cells are cancer cells of epithelial origin. Moreover, the mRNA and protein expression levels of MMP14 in SCLs were higher than those in A549 cells (Figures 1 and 2A), leading to a higher potential for the degradation of the extracellular matrix by the SCLs, which might explain the results of the three-dimensional culture experiments. In fact, there was a previous report that described that the MMP-14 expression level was strongly related to the formation of three-dimensional structures by malignant cells [22].

The Western blot analysis demonstrated that the protein expression levels of MMP-2 and MMP-14 did not decrease, but were actually slightly increased after the batimastat treatments (Figure 2), indicating that, as expected based on its putative mechanism of action, batimastat could suppress only the activities of MMPs, rather than affecting their protein expression levels. In addition, the positive feedback induced by the blockade of the activity of MMPs might have led to the increased expression of MMP-2 and MMP-14 in these cultures.

In the present study, batimastat could suppress the development of subcutaneous tumors in Icr/scid mice (Figure 7), supporting the previous reports showing the effectiveness of this reagent for controlling the growth of other tumors, such as ovarian carcinoma [23], breast cancer [24], pancreatic cancer [17], a human colon cancer model [25] and a metastatic human colon carcinoma model [15]. However, the details of the fundamental mechanisms by which batimastat prevents tumor growth remain to be elucidated. In this study, batimastat could suppress the enlargement of SCL tumors and maintain the weight of mice. It is known that maintenance of the body weight by patients with malignant tumors contributes to a better quality of life [26]. Therefore, it was supposed that both the tumor growth suppression and the effect on the mouse body weight were important for the anti-cancer effects of batimastat.

The findings from this study and the previous study [5] suggest that the behavior and the characteristic of SCLs resemble those of pulmonary intimal sarcoma cells. Clinically, intimal sarcoma is an extremely rare disease, and to the best of our knowledge, there have been no cell lines isolated from intimal sarcoma tissues. Pulmonary intimal sarcoma is believed to originate from the mesenchymal cells present within the intimal layer of pulmonary arteries [27]. Vasuli et al. previously described the immunohistochemical characteristics of pulmonary intimal sarcoma tissues, indicating that the cells of intimal sarcoma were positive for the mesenchymal cell marker, vimentin, negative for the endothelial cell markers von-Willebrand factor (VWF) and CD31, and for the smooth muscle cell marker, α-smooth muscle actin [28]. In addition, this study also described that the undifferentiated intimal sarcoma cells were positive for mesenchymal stem cell markers, such as RUNX-1 or CD44 [28]. As described above, the SCLs in the present study were positive for vimentin and CD44 and negative for CD31 and VWF, α-smooth muscle actin and desmin (Figure 3). SCLs had some characteristics which resemble those of pulmonary intimal sarcoma. However, SCLs were derived from only one patient with CTEPH and the isolation of SCLs was not reproducible. CTEPH and pulmonary initimal sarcoma are completely different diseases and it remains unknown how and why this only one cell line represents characteristics of initimal sarcomas.

In conclusion, this study suggested that MMPs had critical roles on the pathological activities of SCLs and that batimastat might have anti-proliferative and anti-invasive effect on these cells.
