# Correction: The Role of Matrix Metalloproteinase in the Intimal Sarcoma-Like Cells Derived from Endarterectomized Tissues from a Chronic Thromboembolic Pulmonary Hypertension Patient

**DOI:** 10.1371/journal.pone.0091270

**Published:** 2014-02-28

**Authors:** The PLOS ONE Staff

The name of the fourth author is incorrect. The correct name is: Masashi Kantake.

The correct citation is: Jujo T, Sakao S, Tsukahara M, Kantake M, Maruoka M, et al. (2014) The Role of Matrix Metalloproteinase in the Intimal Sarcoma-Like Cells Derived from Endarterectomized Tissues from a Chronic Thromboembolic Pulmonary Hypertension Patient. PLoS ONE 9(1): e87489. doi:10.1371/journal.pone.0087489.

The correct abbreviation of the fourth author's name in the Author Contributions statement is: MK.
